# A Portrait of SARS-CoV-2 Infection in Patients Undergoing Hematopoietic Cell Transplantation: A Systematic Review of the Literature

**DOI:** 10.3390/curroncol29010030

**Published:** 2022-01-13

**Authors:** Adrian J. M. Bailey, Aidan M. Kirkham, Madeline Monaghan, Risa Shorr, C. Arianne Buchan, Christopher Bredeson, David S. Allan

**Affiliations:** 1 Faculty of Medicine, University of Ottawa, Ottawa, ON K1H 8L6, Canada; abail082@uottawa.ca (A.J.M.B.); aikirkham@ohri.ca (A.M.K.); abuchan@toh.ca (C.A.B.); cbredeson@toh.ca (C.B.); 2 Clinical Epidemiology, Ottawa Hospital Research Institute, Ottawa, ON K1H 8L6, Canada; m.monaghan@saba.edu; 3 Medical Library and Learning Services, The Ottawa Hospital, Ottawa, ON K1H 8L6, Canada; rshorr@toh.ca; 4 Division of Infectious Disease, Department of Medicine, University of Ottawa, Ottawa, ON K1H 8L6, Canada; 5 Transplant & Cellular Therapy in the Department of Medicine, The Ottawa Hospital, Ottawa, ON K1H 8L6, Canada

**Keywords:** COVID-19, systematic review, meta-analysis, hematopoietic cell transplant, mortality, treatment

## Abstract

The management of COVID-19 in hematopoietic cell transplant (HCT) recipients represents a special challenge given the variable states of immune dysregulation and altered vaccine efficacy in this population. A systematic search (Ovid Medline and Embase on 1 June 2021) was needed to better understand the presenting features, prognostic factors, and treatment options. Of 897 records, 29 studies were identified in our search. Most studies reporting on adults and pediatric recipients described signs and symptoms that were typical of COVID-19. Overall, the mortality rates were high, with 21% of adults and 6% of pediatric HCT recipients succumbing to COVID-19. The factors reported to be associated with increased mortality included age (HR = 1.21, 95% CI 1.03–1.43, *p* = 0.02), ICU admission (HR = 4.42, 95% CI 2.25–8.65, *p* < 0.001 and HR = 2.26, 95% CI 1.22–4.20, *p* = 0.01 for allogeneic and autologous HCT recipients), and low platelet count (OR = 21.37, 95% CI 1.71–267.11, *p* = 0.01). Performance status was associated with decreased mortality (HR = 0.83, 95% CI 0.74–0.93, *p* = 0.001). A broad range of treatments was described, although no controlled studies were identified. The risk of bias, using the Newcastle–Ottawa scale, was low. Patients undergoing HCT are at a high risk of severe morbidity and mortality associated with COVID-19. Controlled studies investigating potential treatments are required to determine the efficacy and safety in this population.

## 1. Introduction

Severe acute respiratory syndrome coronavirus 2 (SARS-CoV-2), responsible for Coronavirus Disease 2019 (COVID-19), is a zoonotic viral pathogen that has become a widespread global health concern [1,2]. COVID-19 mortality rates are particularly high in individuals who are elderly, have pre-existing comorbidities, and/or are immunocompromised [2]. Hematopoietic cell transplantation (HCT) is potentially curative for many patients with hematological malignancies [3,4] but induces marked immunosuppression during and after transplantation [3,5]. This is particularly true in allogeneic HCT where systemic immune suppression is needed to prevent and/or treat graft versus host disease (GVHD). Many HCT patients, therefore, remain at an increased risk of infections that extend beyond the initial cytopenic period prior to the initial engraftment. In the context of COVID-19, where deferral of a transplant is not often possible and where HCT patients are unlikely to be vaccinated in the immediate peri-transplant period and/or have a compromised response to vaccination [6], recipients likely remain at a greater risk of adverse outcomes if infected [7].

COVID-19 has many well-documented symptoms, such as fever, cough, and fatigue [1], which can be used to recognize, isolate, test, and treat patients who may have contracted the virus [8]. However, many people with COVID-19 remain asymptomatic [9]. While the testing and screening of patients before HCT is commonly performed, it remains possible for patients undergoing HCT to acquire SARS-CoV-2 and develop symptoms of COVID-19 [10]. To provide optimal care for patients and maintain access to transplanting through fully functional programs and services at transplant centers, it is necessary to have an up-to-date understanding of the clinical manifestations in this vulnerable patient population. This will facilitate timely diagnosis and optimal management. While risk factors such as advanced age, obesity, diabetes, and hypertension have been associated with an increased risk of mortality in those with COVID-19, there is less certainty or refinement to our understanding of the clinical manifestations, outcomes, and mortality rates of COVID-19 infection in HCT recipients. Most reports to date are from single centers in isolated jurisdictions that provide a comprehensive description of cases from the early cytopenic phase after transplant, and at later time points, in the context of immune suppression for graft versus host disease or comparing adult to pediatric populations. One recent large review of data from the CIHCTR registry summarizes a larger number of cases of HCT recipients who are more remote from their transplantation date [11]. In this report, we provide the results of a systematic review that summarizes all published articles, allowing us to develop a broader, more comprehensive, and up-to-date portrait of the clinical manifestations and outcomes of HCT recipients contracting COVID-19 at all stages following transplant, including both pediatric and adult recipients. Moreover, we highlight the predictors of mortality and provide an overview of the treatment options.

## 2. Methods

This systematic review was conducted according to the Preferred Reporting Items for Systematic Reviews and Meta-Analyses (PRISMA) guidelines [12]. The protocol of this systematic review was preregistered on the PROSPERO registry (https://www.crd.york.ac.uk/prospero/ (accessed on 17 November 2021), protocol: CRD42020206552, registered 12 November 2020), which was prepared according to the Preferred Reporting Items for Systematic Reviews and Meta-Analyses (PRISMA) Protocol checklist [13].

### 2.1. Literature Search Strategy

In collaboration with a medical information specialist with expertise in systematic review searches, a comprehensive literature search strategy was developed and used to search Medline and Embase from inception to 1 June 2021, without language or date restrictions, using keywords “coronavirus”, “transplant”, and “stem cell”. To ensure all relevant articles were captured, the reference lists of the retrieved studies and relevant reviews were searched. The full search strategy is displayed in Table S1.

### 2.2. Eligibility Criteria

All English-language articles on the clinical characteristics of HCT recipients diagnosed with COVID-19 were included. Both studies of adult and pediatric HCT patients were included. The eligible article types included case reports, case series, prospective and retrospective cohort studies, single-arm studies, randomized controlled trials, correspondences, and editorials. Given the number of large studies on adult HCT populations, only studies with >15 patients were included. Review articles, commentaries, guidelines, and other study types, such as preclinical studies, were excluded.

### 2.3. Study Selection

All unique records identified in the search were uploaded onto Rayyan (rayyan.qcri.org (accessed on 17 November 2021), a systematic review software developed by Hamad Bin Khalifa University. The titles and abstracts of all the records were reviewed by two reviewers (A.B. and A.K.) in an independent and blinded fashion, and all potentially relevant citations’ full texts were assessed for final eligibility. Any discrepancies were resolved through discussion with a third senior author (D.A.).

### 2.4. Data Extraction

Data from study texts, tables, and figures were extracted by two independent reviewers using a standardized data extraction form in Excel (Microsoft Corporation, Seattle, WA, USA). Extracted data included study characteristics (e.g., authors, publication year, study design, and country of study origin); study population (e.g., age, gender, disease, and transplant specifics); and clinical characteristics (symptoms, clinical course, complications, treatment interventions, and cause of death). Any discrepancies were resolved through discussion with a third senior author (D.A.).

### 2.5. Risk of Bias

The risk of bias was assessed using the Newcastle–Ottawa scale for all case–control and cohort studies. An overall rating of 3 stars was interpreted as a high or unclear risk of bias, 4–6 stars as a moderate risk of bias, and 7–9 stars as a low risk of bias.

### 2.6. Meta-Analysis

All meta-analyses were performed using Review Manager version 5.4 (Cochrane Collaboration). Analysis was undertaken when two or more studies reported on the same outcome, with the results of each meta-analysis being interpreted according to the published guidelines. Risk ratios and Odds ratios were used to combine outcomes across studies according to the methods detailed in the Cochrane Handbook for Systematic Reviews of Interventions (version 6). All data were presented with 95% confidence intervals (CI). A descriptive analysis of the studies was carried out if the studies provided insufficient data for inclusion in a meta-analysis.

The I^2^ statistic was used to measure the heterogeneity, and the χ^2^ test was used to determine the homogeneity. I^2^ ranges of <30%, 30–60%, 60–90%, and 90–100% were interpreted as low, moderate, substantial, and critical heterogeneity, respectively. *p* < 0.05 was considered significant for all statistical tests.

## 3. Results

**Figure 1:**
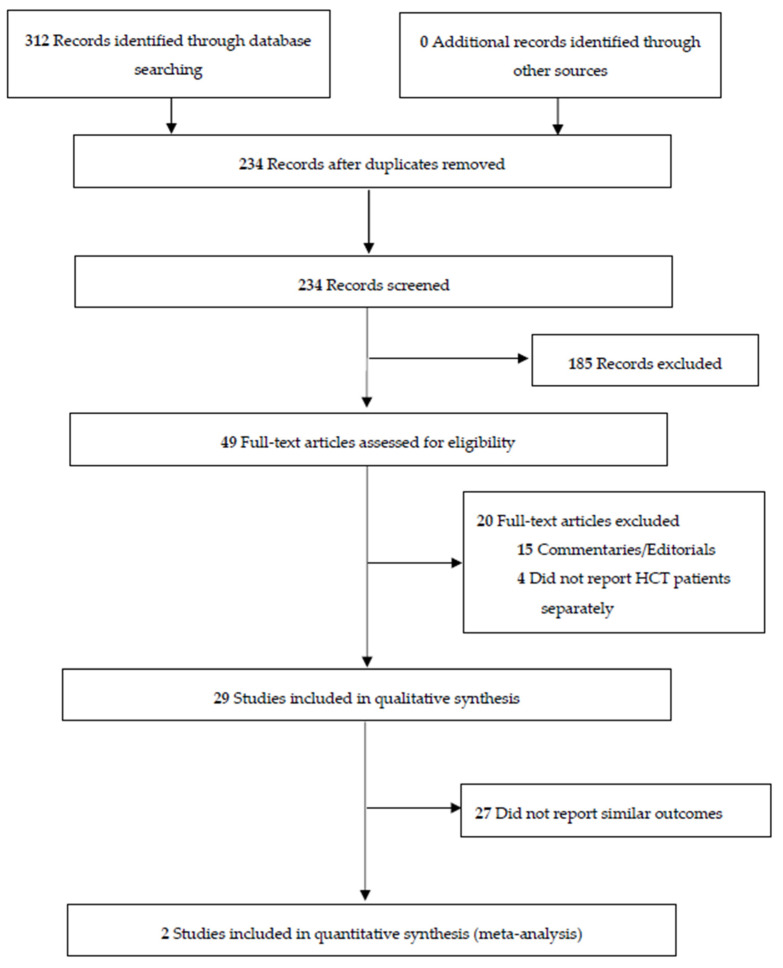
PRISMA flow diagram for the study selection process.

Our systematic search identified 234 unique articles (Figure 1). After the titles and abstracts of these articles were reviewed, 190 articles were excluded, and the full texts of 44 articles were reviewed for final eligibility. A total of 29 articles were included in this systematic review [14,15,16,17,18,19,20,21,22,23,24,25,26,27,28,29,30,31,32,33,34,35,36,37,38,39,40,41,42]. Eleven studies on cases of COVID-19 in adult patients (Table 1) and 18 studies on cases of COVID-19 in pediatric patients were included (Table 2).

### 3.1. Clinical Characteristics

#### 3.1.1. Adult Population

A total of 1285 adult HCT recipients were described in 11 studies [14,15,16,17,18,19,20,21,22,23,24]. The age range was 18–82 years, and 779 (61%) were male. On average, the patients were 18.9 months post-HCT (range −0.9 to 293 months) before their diagnosis with COVID-19. A total of 521 patients (40.5%) were recipients of autologous HCT, 758 (59.0%) were recipients of allogeneic HCT, and 6 (0.5%) received CAR T-cell therapy. Of the studies that reported GVHD history, 320 out of 610 allogeneic HCT patients (52.5%) were reported to have a history of GVHD. It is unclear how many of these patients were receiving immune suppression. Of the studies that reported symptoms (a total of 822 patients), the most common included 538 patients with fever (65%), 416 with cough (51%), 255 with fatigue (31%), 119 with myalgias (14.5%), and 101 with diarrhea (12%). Other principal symptoms that were reported less often included shortness of breath, nausea/vomiting, and headache. It is unclear how many patients were diagnosed by a screening of asymptomatic patients; however, 61 out of 624 (9.8%) patients were asymptomatic in studies that specifically reported on this issue. Of the studies that reported the results of imaging, 82 out of 125 patients (64.8%) had findings (i.e., infiltrates and/or consolidation) on CXR, and 44 out of 58 patients (75.9%) had findings (i.e., infiltrates and/or ground glass opacities) on CT. The overall proportion of adult HCT patients who died was 270 out of 1237 patients (21%). The studies did not report specifically on patients who were diagnosed early after transplant during the cytopenic phase prior to initial engraftment.

#### 3.1.2. Pediatric Population

In 18 studies [25,26,27,28,29,30,31,32,33,34,35,36,37,38,39,40,41,42] that reported on 54 pediatric HCT recipients, the age range was 0.6–17 years. Of the studies that reported sex (total 30 patients), 19 patients (63%) were male. Patients received HCT between 0 and 41 months prior to infection with COVID-19. All patients underwent allogeneic HCT, and 11 out of 21 patients (52%) had a reported history of GVHD. Of 30 patients, common symptoms across the studies included 17 patients with fever (57%), 8 with cough (27%), 3 with rhinitis (10%), 3 with diarrhea (10%), and 2 with sore throat (7%). Of the studies that reported imaging (total 18 patients), 17 out of 18 patients (94%) had findings on CXR or CT, with the most common imaging finding being ground-glass opacities on CT. The overall mortality rate of all the pediatric HCT patients was three out of 54 patients (6%), with seven out of 21 patients (33%) requiring ICU admission.

In total, only four reports described a total of five pediatric patients who were diagnosed with COVID-19 within 2 months of HCT [30,32,36,40]. Three out of four patients underwent imaging that reported positive changes associated with COVID-19, such as ground-glass opacities. Two patients were admitted to the ICU, and one patient died.

### 3.2. Predictors of Mortality Reported in Identified Studies

#### 3.2.1. Age

In total, three studies investigated the association of age and mortality. Across the cohort studies, one study [19] (*n* = 382) found in their multivariate analysis that age was associated with increased mortality across all HCT patients (10-year continuous effect HR 1.21, 95% CI 1.03–1.43, *p* = 0.02), as well as in allogeneic recipients (10-year continuous effect HR 3.17, 95% CI 2.00–5.01, *p* < 0.001) and autologous recipients (HR 2.26, 95% CI 1.22–4.20, *p* = 0.01). Another study [22] performing a multivariate analysis reported an increased risk of mortality among allogeneic HCT recipients who were ≥50 years of age compared to <50 years (*n* = 184) (HR 2.53, 95% CI 1.16–5.52, *p* = 0.02) but no significant difference between age groups in autologous recipients (*n* = 134) (HR 3.31, 95% CI 0.77–14.19, *p* = 0.11). When these two studies were combined in a meta-analysis, age was associated with an increased risk of mortality in both allogeneic (HR 2.99, 95% CI 2.02–4.44, *p* < 0.001) (Figure S1) and autologous (HR 2.40, 95% CI 1.35–4.24, *p* = 0.003) (Figure S2) HCT recipients. One case–control study [24] found increased odds of mortality in their upper quartile for age (63.7–74.1 years) when compared to their lowest quartile (2.4–35.0 years) (OR 12.81, 95% CI 1.19–137.34, *p* = 0.04).

#### 3.2.2. Sex

Only one study investigated the association between sex and mortality. This cohort study [22] found male sex to be associated with an increased risk of mortality in allogeneic (*n* = 184) (HR 3.53, 95% CI 1.44–8.67, *p* = 0.006) but not autologous (*n* = 134) transplant recipients (HR 3.31, 95% CI 0.77–14.19, *p* = 0.11).

#### 3.2.3. Time from Transplant

In total, three studies investigated the association between time from transplant and mortality. Among the cohort studies, one study [22] found an increased risk in patients receiving allogeneic (*n* = 184) (<1 year versus ≥1 year HR 2.67, 95% CI 1.33–5.36, *p* = 0.005) but not autologous recipients (*n* = 134) (<1 year versus ≥1 year HR 3.31, 95% CI 0.77–14.19, *p* = 0.11) in their multivariate analysis, whereas another study [19] (*n* = 382 patients) did not find an effect of time from transplant in their multivariate analysis. Among the case–control studies, one study [24] (*n* = 54) found increased odds in their univariate analysis (4.5–18.8 years, OR 0.05, 95% CI 0.01–0.73, *p* = 0.03). This study, however, did not perform a multivariate analysis.

#### 3.2.4. Autologous Versus Allogeneic HCT

In total, two studies investigated the association between transplant type and mortality. One cohort study [19] (*n* = 382) did not find an increased risk of mortality in patients receiving allogeneic versus autologous HCT in their univariate (HR 0.95, 95% CI 0.64–1.40, *p* = 0.8) or multivariate analysis. One case–control study [20] (*n* = 123 patients) found increased odds of mortality in patients receiving autologous versus allogeneic HCT in their univariate analysis (OR 1.95, 95% CI 0.43–2.5, *p* = 0.048), although this was not significant in a multivariate analysis. 

#### 3.2.5. Comorbidities

In total, two studies investigated the association between comorbidities and mortality. One cohort study [19] (*n* = 382) did not find an increased risk of mortality in patients with preexisting lung pathology (RR 1.15, 95% CI 0.63–2.09, *p* = 0.7). Another case–control study [24] (*n* = 46 patients), which only performed a univariate analysis, did not find increased odds of mortality in patients with any comorbidity (OR 21.37, 95% CI 0.58–18.34, *p* = 0.18).

#### 3.2.6. Underlying Disease

In total, two studies investigated the association between underlying disease and mortality. One cohort study [19] (*n* = 382) did not find an increased risk of mortality in patients according to underlying disease (AML, ALL versus NHL, Hodgkin’s, and CLL versus Other) in their univariate or multivariate analyses. Another cohort study [22] (*n* = 103) found increased mortality in their multivariate analysis in autologous transplant patients with lymphoma compared to plasma cell disorder or myeloma (HR 2.41, 95% CI 1.08–5.38, *p* = 0.033).

#### 3.2.7. Immunosuppressive Treatment

In total, three studies investigated the association between immunosuppression and mortality. Across the cohort studies, one study [22] did not find an increased risk of mortality in allogeneic (*n* = 136) (HR 1.55, 95% CI 0.74–3.26, *p* = 0.24) transplant recipients receiving immunosuppression within 6 months before COVID-19 diagnosis in their multivariate analysis compared to allogeneic patients not receiving immunosuppression. Another study [19] (*n* = 384) found an increased risk of mortality in patients undergoing nonsteroid immunosuppressive treatment in their univariate analysis (HR 2.18, 95% CI 1.03–4.63) but not in their multivariate analysis. Additionally, this study did not find ongoing steroid immunosuppression to be associated with increased mortality in their univariate analysis. One case–control study [24] (*n* = 46 patients), which only performed a univariate analysis, did not find increased odds of mortality in patients receiving any immunosuppressive treatment (OR 2.93, 95% CI 0.69–12.50, *p* = 0.15). Of note, one cohort study [19] (*n* = 384) found an increased risk of mortality in patients moderate to high on the immunodeficiency scoring index in their multivariate analysis (HR 1.84, 1.02–3.33, *p* = 0.04).

#### 3.2.8. Cytopenias

One cohort study [19] (*n* = 384) found ANC levels >500 to be associated with a decreased risk of mortality (HR 0.49, 95% CI 0.27–0.88, *p* = 0.018) in their univariate analysis but not in their multivariate analysis. One case–control study [24] (*n* = 46), which only performed a univariate analysis, found increased odds of mortality (OR 21.37, 95% CI 1.71–267.11, *p* = 0.01) in patients with a platelet count between 11 and 79 × 10^9^/L compared to patients with a platelet count between 221 and 781 × 10^9^/L.

### 3.3. Other Factors

Studies that addressed the role of ethnicity [22], GVHD [19], the neutrophil/lymphocyte ratio [19], and the lymphocyte/CRP ratio [19] did not report any significant correlations with mortality. One study [19] reported an increased mortality in patients admitted to the ICU for both allogeneic and autologous HCT recipients (HR 4.42, 95% CI 2.25–8.65, *p* < 0.001 and HR 2.26, 95% CI 1.22–4.20, *p* = 0.01 in a multivariate analysis for allogeneic and autologous HCT recipients, respectively). A higher performance status was reported in one study [19] (*n* = 384) to be associated with a decreased risk of mortality in their multivariate analysis (HR 0.83, 95% CI 0.74–0.93, *p* = 0.001) and was observed in both allogeneic and autologous recipients.

### 3.4. Treatment

Both adult and pediatric patients have received a broad range of treatments that are summarized in Table 3. The most common treatment was hydroxychloroquine in both adult (21.3%) and pediatric (27.8%) cases. A relatively small number of patients (11.2% adult and 9.3% pediatric patients) received corticosteroids. Most patients received multiple therapies (Table 1 and Table 2), although 20.9% of adult patients and 55.6% of pediatric patients received no specific therapy.

In a multivariate analysis of 216 patients with hematological malignancies with severe COVID-19, which included 123 patients who underwent autologous or allogeneic HCT, one study [20] found a decreased overall mortality with azithromycin or low-dose corticosteroids treatment (OR 0.42, 95% CI 0.2–0.89 and OR 0.31, 95% CI 0.11–0.87, respectively, *p* = 0.02), whereas the use of hydroxychloroquine did not show significant improvement in the overall mortality (OR 0.64, 95% CI 0.37–1.1, *p* = 0.1). The lack of controlled studies precluded an assessment of efficacy for any of the interventions in HCT-only populations.

### 3.5. Risk of Bias

The quality assessment using the Newcastle–Ottawa scale [43] for the included studies is presented in Table S2. All included studies were assigned seven to nine stars, indicating a low risk of bias

## 4. Discussion

In our systematic review, we provide a comprehensive portrait of the clinical manifestations and outcomes of adult and pediatric HCT recipients diagnosed with COVID-19. Our review highlights the paucity of clinical data on the presentation, outcomes, and treatment of COVID-19 in pediatric HCT patients. Moreover, there is a lack of evidence with regards to optimal treatment strategies in adult HCT patients who contract COVID-19. Taken together, the current literature suggests that patients who have undergone HCT appear to present similarly to patients in the general population—commonly presenting with fever, cough, shortness of breath, and myalgias, among other symptoms—or can be asymptomatic. Early diagnosis after transplant can be associated with additional symptoms, such as diarrhea, in some cases, although the relative contribution from COVID-19 as compared to other transplant-related issues such as treatment-induced toxicity or GVHD-associated symptoms is not clear. The coexistence of typical complications of HCT can complicate the diagnosis of COVID-19 in many instances, especially in the early period after HCT. Additionally, pediatric HCT patients may present differently than adult patients. While fever and cough were common symptoms, rhinorrhea and sore throat were the next most prevalent symptoms in the pediatric patients, rather than dyspnea and fatigue, such as in adults. The overall high mortality rate (21% in adults and 6% in pediatric patients) observed in our analysis highlights the vulnerability of HCT recipients and warrants ongoing vigilance in preventing infection and in the guideline-directed vaccination of these individuals [6]. The ASTCT states that the vaccination of HCT patients could be offered as early as three months to HCT and CAR T-cell recipients to prevent infection and severe disease. Considering many HCT recipients have limited immune protection from vaccination and the growing concern for new variants of COVID-19, an updated and refined understanding of the presentation, prognosis, and treatment of COVID-19 in HCT populations remains of high importance.

Given approximately 10% of HCT recipients with COVID-19 are asymptomatic, transplant physicians should maintain a high level of clinical awareness for the diagnosis of COVID-19 in HCT recipients. This highlights the importance of repeated, routine PCR testing to diagnose these individuals and prevent the spread of COVID-19 in transplant facilities and among HCT patients. Additionally, as most patients manifested the typical findings of bilateral and patchy ground-glass opacities on their chest CT, the use of chest imaging appears highly valuable, particularly to help evaluate the extent of the disease and to aid in the diagnosis. A recent update to a living systematic review of chest CT in the diagnosis of COVID-19 indicated a high sensitivity and moderate specificity in the diagnostic test and should be considered early in the course of illness to aid in determination of the extent of the disease [44]. Whether chest CT findings or other potential markers of disease severity can be used to select and guide therapy remains unclear.

In our search, we identified studies that reported various predictors of mortality. Age was associated with an increased risk of mortality in both autologous and allogeneic HCT recipients. The time from transplant was only associated with mortality in the allogeneic transplant recipients, suggesting patients with a remote history of transplant may remain vulnerable to complications of COVID-19 infection. Recipients of both autologous and allogeneic transplants had high mortality rates that were not significantly different. More insight is needed to better understand the factors associated with mortality in this population. Interestingly, although significant in other patient populations, comorbidities were not found to be a significant predictor of morality in HCT recipients. Additionally, GVHD was not found to be associated with mortality. In contrast, an increased performance status was associated with a decreased risk of mortality in both allogeneic and autologous recipients.

The treatment of COVID-19 remains an area of intense investigation. Our review highlights the breadth of treatments used in patients with COVID-19 following HCT. Some treatments, such as hydroxychloroquine and antiviral therapies, were observed frequently in HCT populations, perhaps due to the attention these treatments received early in the pandemic. Subsequent studies, however, have highlighted the lack of efficacy associated with hydroxychloroquine [20,45,46] and lopinavir/ritonavir [47]. A potential benefit associated with remdesivir is supported by several recently conducted randomized controlled trials [48,49], although the efficacy in patients undergoing HCT remains less certain. In particular, the benefits associated with low-dose corticosteroids appear promising [50,51,52], as one study [20] in this review found these treatments to be associated with greater than 50% decreased odds of overall mortality in a mixed population of HCT and non-HCT patients. Thus, these agents may be an important consideration for treating HCT patients with COVID-19. As the pandemic is ongoing, the treatments given to HCT recipients are likely to change given the application of more standardized therapies in the treatment of COVID-19. Nonetheless, the paucity of literature investigating treatments for COVID-19 in HCT recipients highlights the need for controlled studies to confirm the effectiveness of these therapies in this population.

Lastly, several reports have been described regarding HCT patients that have been diagnosed with COVID-19 in the cytopenic phase prior to initial engraftment. Although these studies did not meet the eligibility criteria for inclusion, they highlight the continued need for stringent, preventative measures at transplant centers to prevent SARS-CoV-2 infections in this vulnerable patient population [10,53,54].

This study has limitations worthy of mention. Most of the included studies were observational without control groups. Furthermore, larger studies often reported data for HCT patients along with non-HCT patients, which limits the specific utility of their data for our purposes. Future HCT specific studies will be insightful and increase our understanding of how COVID-19 impacts this unique population. Additionally, most studies described hospitalized cases of COVID-19, and insight regarding the clinical presentation and outcomes of COVID-19 in ambulatory settings and the outcomes of asymptomatic cases detected through screening require more study. Given hospital contact was avoided during the pandemic when possible to prevent COVID-19 transmission, lab testing and imaging may have been performed more sparingly, limiting a true portrait of their diagnostic value. As for treatments, while some therapies appeared promising, further investigation is required to ascertain their effectiveness and tolerability in patients with HCT, particularly for the prevention of COVID-19 symptoms and worsening severity of the disease. Additionally, there is a paucity of information on the clinical presentation and outcomes of pediatric patients who have undergone HCT. Although our systematic search captured and analyzed all the available literature, future high-quality cross-sectional, case–control, and cohort studies are required to validate the observations made in this review, specifically for pediatric HCT populations. Lastly, some patients in our analysis may be duplicated or may not meet our inclusion criteria, as some studies were larger cooperative studies that may have included patients from single-center studies and/or underwent chimeric-antigen receptor T-cell therapy and/or developed COVID-19 in the weeks prior to HCT. Overall, the impact of these cases is likely small and unlikely to influence our analysis in a significant manner.

Our analysis provides a comprehensive portrait of COVID-19 in HCT recipients and illustrates how typical and more unusual manifestations should prompt early testing with heightened clinical awareness that can facilitate the optimal diagnosis, treatment, and management of outbreaks at transplant centers. Moreover, insight regarding the risk factors associated with mortality in HCT patients with COVID-19 may allow the identification of patients for preventative and/or early therapeutic interventions. However, controlled studies are needed in this population to confirm the effectiveness of these therapies. Consideration for the early vaccination of HCT patients who remain at high risk of mortality from COVID-19 remains an evolving and critical priority, and studies are needed in this population to confirm the efficacy and safety of the vaccines.

## Figures and Tables

**Table 1:** Summary of the studies of adult patients with COVID-19 following HCT.

Study	*n* (Age Range)	Male, *n* (%)	Time Since Transplant, Mean Months (Range)	Graft Type, (%) Auto/Allo/CART	GVHD History, *n* (% of Allos)	ICU (%)	Deaths (%)
(14)	32 (19–74)	25 (78)	NR	20/12/0	NR	7 (22)	5 (16)
(15)	28 (50–67) *	16 (57)	21.9 (11.1–42.5)	12/15/1	7 (47)	7 (25)	5 (18)
(16)	54 (0–73.5)	31 (57)	15.6 (0.3–228)	0/54/0	54 (100)	13 (24)	13 (24)
(17)	113 (34–64)	64 (57)	18 (4–53), Auto 15 (7–37), Allo	42/71/0	NR	9 (8.0)	18 (16)
(18)	91 (NR)	53 (58)	14.9 (16.3–38.9)	39/52/0	12 (23)	13 (14)	4 (4.4)
(19)	33 (27–71)	21 (64)	NR	19/14/0	6 (43)	4 (12)	3 (9.0)
(20)	382 (1–81.6)	236 (62)	24.6 (−0.9–350), Auto 15.8 (0.2–293), Allo	146/236/0	89 (38)	86 (22)	107 (28)
(21)	123 (1–75)	74 (60)	26.3, Auto14.7, Allo	58/65/0	NR	NR	25 (20)
(22)	77 (52–68)	49 (64)	<6 months (*n* = 10) 6–12 months (*n* = 10), 12–36 months (*n* = 27), >36 months (*n* = 30)	37/35/5	17 (49)	NR	17 (22)
(23)	318 (30–65)	188 (59)	23 (8–51), Auto 17 (8–46), Allo	134/184/0	126 (68)	NR	66 (21) **
(24)	34 (24–76)	22 (65)	17.4 (1–248.7)	14/20/0	9 (45)	11 (32)	7 (21)
TOTAL	1285	779 (60.6)	(−0.9–350)	521 (40.5%)/ 758 (59.0%)/ 6 (0.5%)	320/610 (52.5%)	150/767 (19.6%)	272/1285 (21.2%)

CART, chimeric antigen receptor T-cell therapy; GVHD, graft versus host disease; ICU, intensive care unit; NR, not reported; auto, autologous; allo, allogeneic. * Interquartile range and ** unknown status in 48 patients.

**Table 2:** Summary of the studies describing pediatric patients with COVID-19 following HCT.

Study	*n* (Age Range)	Male, *n* (%)	Time Since Transplant, (Months)	Graft	GVHD History, *n* (%)	ICU (%)	Deaths (%)
(25)	3 (NR)	NR	25, 41, 41	NR	NR	0 (0%)	0 (0%)
(26)	1 (0.6)	0	3	Allo	1	NR	0 (0%)
(27)	3 (NR)	NR	5–22	Allo	NR	NR	0 (0%)
(28)	6 (NR)	NR	NR	NR	NR	0 (0%)	0 (0%)
(29)	8 (NR)	NR	NR	NR	NR	NR	2 (25%)
(30)	1 (15)	0	0.3	Allo	0	1 (100%)	0 (0%)
(31)	1 (8)	1	NR	Allo	NR	1 (100%)	0 (0%)
(32)	1 (8)	1	0.7	Allo	0	NR	1 (100%)
(33)	1 (17)	0	3	NR	1	NR	0 (0%)
(34)	1 (5)	1	5	Allo	1	NR	0 (0%)
(35)	2 (2–17)	1	5, 6	Allo	1 (50)	NR	0 (0%)
(36)	1 (9)	0	0	Allo	0	NR	0 (0%)
(37)	2 (5,13)	1	2	Allo	NR	2 (100%)	0 (0%)
(38)	4 (3–10)	3	0.6, 13, 15, 16	Allo	3 (75)	1 (25%)	1 (25%)
(39)	1 (16)	0	5	Allo	1	NR	0 (0%)
(40)	8 (1–12)	7	1–24	Allo	3 (38)	NR	1 (12.5%)
(41)	4 (NR)	NR	NR	NR	NR	2 (50%)	0 (0%)
(42)	6 (1.9–12.6)	4	NR	Allo	NR	0 (0%)	0 (0%)
TOTAL	54	19/30 (63)	0–24	13/13 (100%)	11/21 (52%)	7/27 (26%)	5/54 (9.3%)

GVHD, graft versus host disease; ICU, intensive care unit; Allo, allogeneic; NR: not reported.

**Table 3:** Treatment of COVID-19 in patients following HCT. Note that, in almost all cases, patients received multiple treatments. Therefore, the totals exceed 100%.

Treatment	Adult HCT Patients, *n* (%)	Pediatric HCT Patients, *n* (%)
Hydroxychloroquine (HCQ)	274 (21.3)	15 (27.8)
Azithromycin (AZT)	92 (7.2)	9 (16.7)
Corticosteroids	144 (11.2)	5 (9.3)
Tocilizumab	87 (6.8)	5 (9.3)
Convalescent plasma	42 (3.3)	1 (1.9)
Remdesivir	74 (5.8)	6 (11.1)
Immune globulin *	7 (0.5)	2 (3.7)
Lopinavir/ritonavir	52 (4.0)	3 (5.6)
Ruxilotinib	4 (0.3)	0 (0.0)
Anakinra	20 (1.6)	2 (3.7)
Favipiravir	39 (3.0)	0 (0.0)
Oseltamivir	18 (1.4)	2 (3.7)
Methylprednisolone	14 (1.1)	1 (1.9)
Protease inhibitors	47 (3.7)	0 (0.0)
HCQ + AZT	26 (2.0)	0 (0.0)
HCQ + Lopinavir/Ritonavir	22 (1.7)	0 (0.0)
AZT + Lopinavir/Ritonavir	14 (1.1)	0 (0.0)
HCQ + AZT + Lopinavir/Ritonavir	9 (0.7)	0 (0.0)
Acyclovir or Valacyclovir	23 (1.8)	0 (0.0)
HCQ + Favipiravir	5 (0.4)	0 (0.0)
Siltuximab	3 (0.2)	1 (1.9)
No specific treatment	268 (20.9)	30 (55.6)
Other **	93 ** (7.2)	22 *** (40.7)

* Includes 1 case of hyperimmune SARS immune globulin. ** Includes Others unspecified (40), Antibiotics (21), High dose vitamin C (8), Other unspecified antivirals (4), Interferon-B (4), Eculizumab (3), Baricitinib (2) N-acetyl cysteine (2), Sarilumab (1), Ribavirin (1), and Mesenchymal stem cells (1). *** Includes Meropenem (4), Cotrimoxazole (4), Antifungals unspecified (4), Lopinavir (2), Vancomycin (2), Ceftriaxone, then Cefuroxime (1), SARS-CoV-2Ab (1), Tazocin (1), Heparin (1), Dexamethasone (1), and CD45RA cellular therapy (1).

## Supplementary Materials

The following supporting information can be downloaded at https://www.mdpi.com/article/10.3390/curroncol29010030/s1: Table S1: Search strategy. Table S2: Scoring Distribution of Quality Assessment. Figure S1: Risk of mortality with increasing age in allogenic transplant recipients. Figure S2: Risk of mortality with increasing age in autologous transplant recipients.
