# Supplementary material for: A Portrait of SARS-CoV-2 Infection in Patients Undergoing Hematopoietic Cell Transplantation: A Systematic Review of the Literature
Source: Curr Oncol. 2022 Jan 13;29(1):337–49. doi: 10.3390/curroncol29010030 (PMC8774852; doi:10.3390/curroncol29010030)

Current Oncology

MDPI

Systematic Review

A Portrait of Sars-CoV-2 Infection in Patients Undergoing Hematopoietic Cell Transplantation: A Systematic Review of the Literature

Adrian J.M. Bailey, Aidan M. Kirkham, Madeline Monaghan [2], Risa Shorr, C. Arianne Buchan, Christopher Bredeson and David S. Allan

Table S1. Search strategy for Medline and Embase, updated to June 1st 2021. (Performed by Risa Shorr, Librarian Specialising in Systematic Searches, The Ottawa Hospital).

Ovid MEDLINE(R) ALL <1946 to June 04, 2021>

1 COVID-19/ 82696
2 (exp coronavirus/ or coronavirus*.mp. or corona virus*.mp.) and (wuhan or beijing or shanghai or hubei).mp. 5523
3 ((coronavirus* or corona virus* or coronavirus* or coronaviridae or coronaviridae or betacoronavirus*) adj3 ("19" or "2019")).tw. 36191
4 covid.tw,kw. 121646
5 covid19.tw,kw. or covid 19.kw.55184
6 sars cov 2.tw,kw. 45292
7 (ncov or n cov).tw,kw. 1479
8 (novel coronavirus* or novel corona virus*).tw,kw. 8937
9 (CoV 2 or CoV2 or sarscov2 or 2019nCoV or novel CoV or wuhan virus*).tw,kw. 40460
10 (Coronavirus Infections/ or Severe Acute Respiratory Syndrome/) and (Pandemics/ or pandemic*.tw,kf.) 39658
11 or/1-10 142789
12 Bone Marrow Transplantation/ 44968
13 exp Hematopoietic Stem Cell Transplantation/ 48057
14 bmt.tw,kw. 10737
15 bone marrow transplant*.tw,kw. 33565
16 stem cell transplant*.tw,kw. 51378
17 ((h?ematopoietic adj3 transplant*) or hsct*).tw. 35511
18 (peripheral blood cell transplant* or pbsct).tw,kw. 1446
19 STEM CELL TRANSPLANTATION/ 24666
20 (hsct or H?ematopoietic Stem Cell Transplant*).kw. 3369
21 stem cell therap*.tw,kw. 7878
22 Cord Blood Stem Cell Transplantation/ 3379
23 ((cord blood or placenta* blood*) adj5 transplant*).tw. 4000
24 ((cord blood or placenta* blood*) and transplant*).kf. 636
25 or/12-24 149572

Curr. Oncol. **2022**, *29*, 337–349. https://doi.org/10.3390/curroncol29010030 www.mdpi.com/journal/curroncol

26 11 and 25 340

27 (2021031* or 2021032* or 2021033* or 202104* or 202105* or 202106*).dt. 393807

28 26 and 27 68

Embase Classic+Embase <1947 to 2021 June 04>

1 coronavirus disease 2019/ 117729

2 (Coronavirinae/ or coronavirus*.mp. or corona virus*.mp.) and (wuhan or beijing or shanghai or hubei).mp. 6164

3 ((coronavirus* or corona virus* or coronavirus* or coronaviridae or coronaviridae or betacoronavirus*) adj3 ("19" or "2019")).tw. 34145

4 (covid or covid19).tw. 120084

5 sars cov 2.tw. 37667

6 (ncov or n cov).tw.1524

7 (novel coronavirus* or novel corona virus*).tw. 8411

8 (CoV 2 or CoV2 or sarscov2 or 2019nCoV or novel CoV or wuhan virus).tw. 39128

9 (coronavirus infection/ or severe acute respiratory syndrome/) and (pandemic/ or pandemic*.tw.) 11199

10 limit 9 to yr="2019 -Current" 10428

11 1 or 2 or 3 or 4 or 5 or 6 or 7 or 8 or 10 145891

12 exp hematopoietic stem cell transplantation/ 71589

13 allogenic bone marrow transplantation/ 10239

14 stem cell transplantation/ 44203

15 bone marrow transplantation/ 54499

16 allogeneic stem cell transplantation/ 16270

17 allogeneic peripheral blood stem cell transplantation/ 1192

18 peripheral blood stem cell transplantation/ 3425

19 allogeneic hematopoietic stem cell transplantation/26050

20 ((h?ematopoietic adj3 transplant*) or hsct*).tw. 68614

21 (peripheral blood cell transplant* or pbsct).tw. 2296

22 stem cell transplant*.tw. 97767

23 bone marrow transplant*.tw. 44082

24 stem cell therap$.tw. 10258

25 Cord Blood Stem Cell Transplantation/ 6358

26 ((cord blood or placenta* blood*) adj5 transplant*).tw. 6819

27 CBSCT.tw. 45

28 exp autologous stem cell transplantation/ 27170

29 or/12-28 245191

30 11 and 29 595

31 (2021031* or 2021032* or 2021033* or 202104* or 202105* or 202106*).dc. 695964

32 30 and 31 158

Table S2. Scoring Distribution of Quality Assessment of Studies Using the Newcastle-Ottawa Scale.

STUDY	SELECTION	COMPARABILITY	OUTCOME	TOTAL
		**Cohort Studies**		
Coll 2020	4	1	3	8
Piñana 2020	4	2	3	9
Camargo 2021	4	0	3	7
Shah 2020	4	1	3	8
Xhaard 2021	4	1	3	8
		**Case-Control Studies**		
Sharma 2021	4	2	3	9
Ljungman 2021	4	2	3	9

Figure S1. Risk of mortality with increasing age in allogenic transplant recipients.

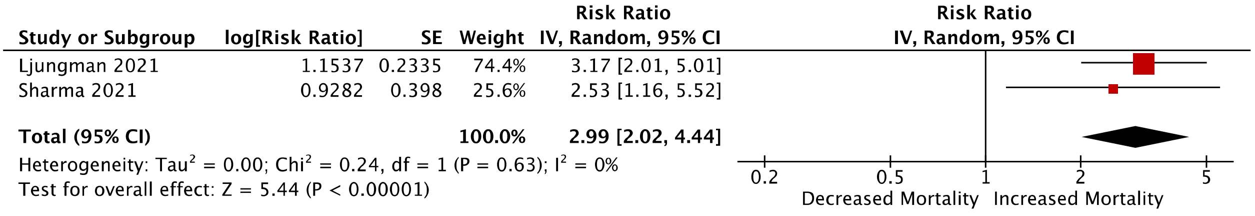

Figure S2. Risk of mortality with increasing age in autologous transplant recipients.

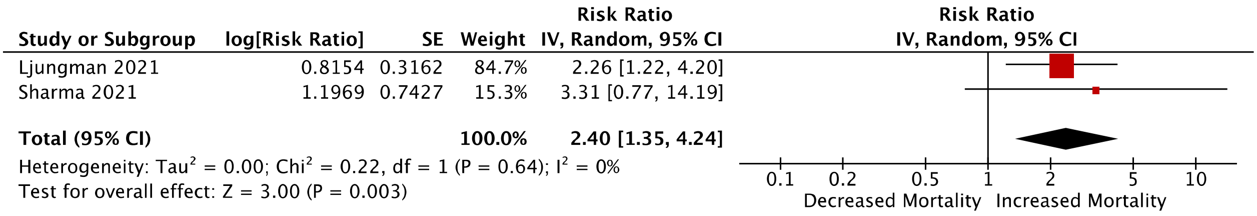

Supplement: Supplementary file 1 [file curroncol-29-00030-s001.zip › curroncol-1493500-supplementary.pdf]
